# Comparative analysis of clinical outcomes cerebellopontine angle meningioma versus cerebellopontine angle schwannoma: A retrospective insight from Pakistan

**DOI:** 10.12669/pjms.41.13(PINS-NNOS).13374

**Published:** 2025-12

**Authors:** Tariq Imran Khokhar, Abdul Ghafoor, Muhammad Shakir, Faiqa Ijaz Khan, Haseeb Mehmood Qadri

**Affiliations:** 1 Dr. Tariq Imran Khokhar, MBBS, FCPS. Punjab Institute of Neurosciences, Lahore, Pakistan; 2 Dr. Abdul Ghafoor, MBBS, FCPS. Punjab Institute of Neurosciences, Lahore, Pakistan; 3 Dr. Muhammad Shakir, MBBS, MS. Punjab Institute of Neurosciences, Lahore, Pakistan; 4 Dr. Faiqa Ijaz Khan, MBBS. Punjab Institute of Neurosciences, Lahore, Pakistan; 5 Dr. Haseeb Mehmood Qadri, MBBS. Punjab Institute of Neurosciences, Lahore, Pakistan

**Keywords:** Acoustic neuroma, Cerebellopontine angle, Cerebellopontine angle tumors, Meningioma, Pakistan, Schwannoma

## Abstract

**Background and Objective:**

Meningiomas follow schwannoma as the second common space occupying lesions (SOL) in the cerebellopontine angle (CPA). Tissue-based differences in outcomes for these neoplasms using retrosigmoid approach have not been studied previously. We aimed to evaluate tumor characteristics and surgical outcomes based on tumor histopathology

**Methodology:**

This retrospective observational study was conducted at the Department of neurosurgery in Punjab Institute of Neurosciences, involving consecutive, single-surgical team led cases of CPA space occupying lesions (SOL) between 2021 and 2024. Chi-square test was used to determine any significant differences between Schwannomas and meningiomas in terms of tumor consistency, vascularity, extent of resection and postoperative complications. Fischer’s exact test was applied when expected cell counts were low.

**Results:**

Of the hundred patients included, 63 were the cases of Schwannoma and 37 were meningioma of CPA. A male preponderance of 57.1% (36) with an overall mean age of 37.7±13.4 years was noted in the Schwannoma group. Contrastingly, 56.8% patients were females with an overall mean age 39.3±13.1 years in the meningioma group. A significant association in occurrence of postoperative facial nerve weakness and loss of gag reflex between Schwannomas and meningiomas was seen i.e., χ²(1)= 6.52 p = 0.007 and χ²(1) = 12.94, p = 0.000, respectively (with significant results for Fischer exact test as well i.e., p<0.05). Gross total resection was achievable in 88.9% (56) cases of Schwannoma and 100% (37) of meningioma, but no significant differences in tumor consistency, vascularity and extent of resection were observed between Schwannomas and meningiomas i.e., χ²(2)= 5.40 p= .067, χ²(3)= 5.89 p= .117 and χ²(2)= 4.42 p= 0.11, respectively.

**Conclusion:**

Schwannomas were more likely to have postoperative facial nerve weakness and loss of gag reflex in comparison to meningiomas. However, there are no significant differences between Schwannomas and meningiomas in terms of tumor consistency, vascularity and extent of resection.

## INTRODUCTION

Cerebellopontine angle tumors comprises 5-10 % of all intracranial tumors, among which most common are vestibular schwannomas (VS) (75-86%) and meningiomas (5-10%).^1^-^3^ Both can simultaneously occur in NF2.^4^ Schwannomas of basal cranial nerves (0.7 %) and trigeminal nerve (1%) are also encountered.^5^,^6^ CPA tumors have predilection towards female 92%.^7^ VS presents with sensorineural type hearing loss or vestibular dysfunction.^3^ In contrast to meningioma which presents with hearing loss, trigeminal nerve dysfunction signs (up-to 49%) and cerebellar signs (25-52%),^3^ other includes facial weakness, tongue numbness or weakness, dysarthria and dysphagia.^7^,^8^

For VS, trans-labyrinthine, retro-labyrinthine, middle cranial fossa and retrosigmoid, has been documented in literature, whereas in addition to aforementioned transpetrosal has been mentioned for meningiomas, depending upon the tumor size, origin and pre-op neurology.^3^,^8^,^9^ Retrosigmoid is now favored by most surgeons in cases of preserved serviceable hearing, and where tumor extend towards jugular foramen, because of better exposure.^1^,^7^,^8^ But it also results in basal nerve palsies and cerebrospinal fluid (CSF) leak as compared to trans-labyrinthine.^3^,^9^ Facial nerve preservation (21%) is also seen in retrosigmoid as compared to trans-labyrinthine (38%) approach.^8^

However histopathological diagnosis compared with post-operative outcome is infrequently studied. VS are intimately involved with CN VIII and hence the VII-VIII complex are more prone to impairment.^9^ However in meningiomas post-operative complications (loss of gag reflex (5.9%), CN VII palsy (5.9%) and hearing loss (0%)) can be attributed to its tough nature and its attachment to dura at different sites.^9^

Surgical outcomes of patients undergoing retrosigmoid approach for cerebellopontine angle (CPA) schwannomas and meningiomas, in relation to the tumors’ morphological and structural features, have not been previously studied in Pakistan. This is the first study to compare these features between CPA schwannomas and meningiomas and correlate them with postoperative outcomes.

## METHODOLOGY

This was retrospective observational study, conducted in Department of Neurosurgery, Punjab Institute of Neurosciences, upon the patients who underwent cerebellopontine angle excision from January 2022 to December 2024. Non-probability consecutive type sampling technique was used. All surgeries were done by the same surgical team, in elective neurosurgical operating rooms (ORs) at PINS.

### Ethical Approval:

Study was approved by Institutional Review Board Punjab Institute of Neurosciences, with approval number 2036/IRB/PINS/Approval/2025 dated: January 23, 2025.

### Inclusion criteria:

- All patients with age above 14 years, who underwent retrosigmoid craniectomy for cerebellopontine angle tumor (meningioma or schwannoma), with at least three months of follow-up, were included in this study. Regardless of the neoadjuvant therapy or presenting features all patients with the preserved data were included.

### Exclusion criteria:

- Patients who lost follow up and whose data was incomplete were excluded from the study.

### Author-proposed Operational Definitions:

- All patients underwent pre-operative MRI and post-operative CT scan, to estimate the extent of resection. Tumors dimension and visibility on imaging were assessed. Gross total resection (GTR) was defined as surgical resection of >95% of tumor, near-total resection as >90%, subtotal resection as >70% and partial resection >50%.
- Vascularity of tumor was defined as following. Mild: bleed controlled with 1-2 cottonoids. Moderate: needing more than > 4-5 cottonoids and irrigation. Severe: need aggressive management, cauterization along with the need of anesthetist to follow-up.
- Consistency was assessed manually when chunk of tumor was removed. Soft: felt like ear lobule. Firm: felt like cartilage. Hard: felt like bone.

Data was retrieved from patient files records and Picture archiving and communicating system (PACS) in one month. Data was collected via Google form. Study focused on patient’s pre-op complaints, Glasgow Coma scale (GCS), Karnofsky Performance status (KPS) score, intraoperative tumor features (consistency and vascularity), CSF diversion, radiological findings, histopathological diagnosis and postoperative outcomes.

### Data analysis technique:

Data was analyzed using percentages, frequencies, measures of central tendencies (mean, mode, median) and central dispersion (standard deviation). Chi-square test was used to determine any significant differences between schwannomas and meningiomas in terms of tumor consistency, vascularity, extent of resection and postoperative complications. Fischer’s exact test was applied when expected cell counts were low.

## RESULTS

Total of one hundred patients were included in this study of which. 63% (63) cases were of schwannoma and 37% (37) were meningiomas. Mean age in schwannoma group was 37.7 ± 13 SD (Table-I). Symptoms on presentation, GCS, KPS and need for CSG diversion are mentioned in (Table-I). None of the patients however received neo-adjuvant therapy of any kind.

**Table-I:** Descriptive analysis of the demographics and clinical presentation of patients with CPA Schwannoma versus CPA meningioma.

Variables	Schwannoma	Meningioma
Patient distribution, % (n)	63(63)	37(37)
Age, mean ± SD (years)	37.7±13.4	39.3±13.1
***Gender distribution, M/F, % (n)***		
Female	42.9 (27)	56.80(21)
Male	57.1 (36)	43.20(16)
***Symptoms at Presentation, % (n)***		
Headache	88.90(56)	100(37)
Hearing Loss	77.80(49)	54.10(20)
Vomiting	20.60(13)	18.90(7)
Facial weakness	14.30(9)	2.70(1)
Visual disturbance	14.30(9)	2.70(1)
Vertigo	6.30(4)	27.00(10)
Gait disturbance	6.30(4)	8.10(3)
***Pre-operative GCS, % (n)***		
3-8	0(0)	0(0)
9-12	1.60(1)	0(0)
13-15	98.40(62)	100(37)
Pre-operative KPS, mean ± SD	79.2±13.8	90.5±5.2
Need for pre-operative CSF diversion? % (n)	14.30(9)	8.10(3)

On imaging 92.1 % (58) schwannomas were heterogeneously contrast enhancing compared to 81.1% (30) meningiomas which were homogeneously enhancing. 13.5 % (5) of meningiomas occupied two compartments. And majority of them were on right side 83.8 % (31) (Table-II).

**Table-II:** Descriptive analysis of pre-operative neuroimaging of patients with CPA schwannoma versus CPA meningioma.

Variables	Schwannoma	Meningioma
***MRI T1 Findings, % (n)***		
Hypointense	74.60(47)	24.30(9)
Isointense	25.40(16)	75.70(28)
***MRI T2 Findings, % (n)***		
Isointense	0(0)	32.40(12)
Hyperintense	100(63)	67.60(25)
***MRI Contrast Findings, % (n)***		
Non Enhancing	4.80(3)	0(0)
Homogeneous	3.20(2)	81.10(30)
Heterogeneous	92.10(58)	18.90(7)
***Site of SOL, % (n)***		
Posterior cranial fossa	98.40(62)	86.50(32)
Middle and Posterior cranial fossa	1.60(1)	13.50(5)
***Side of SOL, % (n)***		
Right	47.60(30)	83.80(31)
Left	47.60(30)	16.20(6)
Bilateral	4.80(3)	0(0)

Retrosigmoid craniotomy was used in all cases. None of our patients had post-operative CSF leak. 38.1 % (24) from schwannoma and 5.4 % (2) from meningioma group had complete loss of gag reflex and required PEG tube (percutaneous endoscopic gastrostomy). Gross total resection (GTR) was achieved in 88.9 % (56) of schwannoma compared to 100 % (37) of meningiomas. Schawannomas were mostly soft 76.3 % (48) and moderately vascular 66.7 % (42) (Table-III).

**Table-III:** Descriptive analysis of parameters of surgical management and post-operative sequelae of patients with CPA schwannoma versus CPA meningioma.

Variables	Schwannoma	Meningioma
***Type of Surgical Approach, % (n)***		
Retrosigmoid Craniectomy	100(63)	100(37)
***Consistency, % (n)***		
Soft	76.20(48)	59.50(22)
Firm	9.50(6)	27.00(10)
Hard	14.30(9)	13.50(5)
***Vascularity, % (n)***		
Mild	15.90(10)	32.40(12)
Moderate	66.70(42)	48.60(18)
High	17.50(11)	16.20(6)
Avascular	0(0)	2.70(1)
***Extent of Resection, % (n)***		
GTR	88.90(56)	100(37)
NTR	1.60(1)	0(0)
STR	9.50(6)	0(0)
***Post-operative GCS, % (n)***		
3-8	0(0)	0(0)
9-12	19.00(12)	2.70(1)
13-15	81.00(51)	97.30(36)
***Post-operative Complications, % (n)***		
Facial nerve weakness	15.90(10)	0(0)
Absent gag reflex	38.10(24)	5.40(2)
CSF leak	0(0)	0(0)
Post-operative KPS, mean ± SD	76.3±15.4	88.6±10.8
Need for post-operative CSF diversion, % (n)	Nil	13.50(5)

GTR: gross total excision, NTR: near total excision, STR: subtotal excision, KPS: Karnofsky Performance Status.

Schwannomas were more likely to have postoperative facial nerve weakness and loss of gag reflex in comparison to meningiomas i.e., χ²(1)= 6.52 p = 0.007 and χ²(1) = 12.94, p = 0.000 respectively (with significant results for Fischer exact test as well i.e., p<0.05). The results revealed a significant association in occurrence of postoperative facial nerve weakness and loss of gag reflex between schwannomas and meningiomas. On the other hand, no significant differences in tumor consistency, vascularity and extent of resection were observed between schwannomas and meningiomas i.e., χ²(2)= 5.40 p = .067, χ²(3)= 5.89 p = .117 and χ²(2)= 4.42 p = 0.11 respectively.

## DISCUSSION

Cerebellopontine angle is a triangular space in front of cerebellum, lateral to pons and inferior to the tentorium. It harbors CN V, VI, VII, VIII, SCA (superior cerebellar artery), AICA (anterior inferior cerebellar artery), choroid plexus and it is in close proximity with brainstem. 3 Amongst CPA tumors vestibular schwannoma has the higher incidence of 75-86%, followed by meningioma 5-15%.^3^ Tumors distribution (63% VS and 37% meningioma) in our results was in close approximation with literature, and a slight deviation can be attributed limited number of cases.

Babu et al. reported median age of 55 year in VS, peaking in 6^th^ decade.^10^,^11^ Our mean age of 37.7±13.4 SD might be due to early presentation, owing to the low functional status. Our mean KPS in VS group was 79.2±13.8 SD compared to Spena et al. who reported the KPS of > 90 in 90.3% of patients and thus the mean age in their study was 55 years in his study.^12^ Female predominance was found in literature, 51.98%, which was also slightly drifted in our present study, where we encountered 57.1 % (36) male patients.^10^,^11^,^13^ In meningioma group our results showed the mean age of 39.3±13.1 SD and female predominance of 56.8% (21) consistent with the literature. Magill et al. outlined the median age of 62 years (ranging from 37 to 90 years) and amongst 80% (41) were females.^14^ Meta-analysis done by Qi ZY et al. showed the association between the risk of meningioma in hormonal replacement therapy (HRT), post-menopausal and increase number of births.^15^ Meningiomas display progesterone (88%) and estrogen (40%) receptors, this is hypothesized to be the rationale behind its prevalence in females.^16^

In both groups, most common presenting symptom in our study was headaches followed by hearing loss, which was in accordant with the existing literature.^12^,^14^,^15^ Amongst cranial nerve deficit hearing loss is often the bothering symptom that leads to the patient’s presentation in VS cases.^3^ Schwannomas arises from the nerve sheath of peripheral nerves, CPA is hub of cranial nerves and in that way the nerve specific symptoms are often profound. Meningiomas tends to cause ataxia (46%) and vertigo more frequently than VS (gait disturbance in 29%).^12^,^16^ They subtly grows along the petroclival dura or tentorium thereby compressing cerebellum causing cerebellar signs as well as contralateral long track signs.^3^,^12^,^16^ It is said that, post fossa tumors tends to obstruct the CSF outflow tracks, resulting in hydrocephalus. In our study pre-operative CSF diversion was needed in 14.3 % (9) cases of VS and only 8.1% (3) of meningiomas. Close proximity of basal CN with brainstem, hence their tumors, block the CSF flow at the level of 4^th^ ventricle.^3^ To our knowledge this is the first study to report GCS in CPA comparison study (13-15 in majority of our cases).

VS appears iso-hypointense on T1 compared to meningiomas who are iso-hypointense (mostly Isointense) on T1.^3^ On T2 both looks hyperintense, meningioma a slight more hyper than VS.^3^ However we found 32.4 % (12) meningiomas isointense. Histopathological different types can be the basis of this finding but in this study unfortunately we did not had those details. In 2007 Bonneville et al. reported VS contrast enhancement 50-60% homogeneously, 30-40% heterogeneously and 5-15% with cystic component.^17^ VS can be intracanalicular or intracisternal or dumbbell shape comprising both compartments.^17^ Intracisternal can compress the cerebellum and brainstem, making itself symptomatic early. Intracanalicular can widen the internal auditory canal (IAC) on imaging. In our case 92.1 % were heterogeneously enhancing. Meningiomas on the other hand shows avid homogenous enhancement in congruous with our results.^18^ In our study 13.5 % meningiomas tends to occupy more than one cranial fossa (posterior and middle). They arise from dura hence have broad based dural attachment over the petrous bone.^18^ They can grow into the petrous bone, larger meningiomas tends to occupy not only middle fossa also they can extend to sphenoid wing, cavernous sinus and suprasellar region.^19^ About 83.8% (31) meningiomas in our case were right sided consistent with literature.^19^ Inskip et al. found a slight predominance of meningiomas on left side VS on right side, however more data is required in this area to hypothesize an association.^20^

All of our patients had undergone retrosigmoid (RS) craniectomy. Gross total resection (GTR) was achievable in 88.9% (56) of VS and 100% (37) in meningioma cases. Rizk et al. reported 65% soft and 35 % firm VS, additionally facial function was better in soft tumors.^21^ 76.2 % (48) VS in our study were soft but, 15.9 % (10) had post-operative facial dysfunction. Adhesion of tumor with VII-VIII complex or in cases of other schwannomas, the thinning and stretch on the nerve makes them more prone to harm.^22^ Schwannomas are sometimes called VAFE tumors (vascular, adhesion, fibrous and engulfing) and a thin layer of tumor is left in order to preserve nerve function.^22^ Meningiomas were 59.5 % (22) soft and only 13.5 % (5) were hard, variance in histopathological types can account for variability in consistency of tumor. Little et al. reported reduced percentage of GTR in fibrous consistency tumors, with higher (41%) rates of post-operative CN deficit.^23^ Fibrous tumors are known to be hard and difficult to excise. And are, hence prone to cause more damage to the nearby structures. Our tumors were mostly soft hence we achieved GTR in maximum case. However, in fibrous vs. non-fibrous, vascular vs. avascular, engulfing vs. sparing, tumors no statistically significant difference was found in terms of post-operative outcomes.^23^,^24^ Most advance centers use, intra-operative neuro-monitoring (IONM) for preservation of cranial nerve. This can be done in the form of auditory brainstem response (ABR) potential or facial nerve monitoring. No use of IONM also poses one of the limitations in our current study.

Awan et al. reported 65% post operated patients of VS having facial nerve palsy.^25^ We also had maximum number of CN palsies in VS group compared to meningioma. 38.1% (24) patients of VS group had complete loss of gag, resulting in placement of PEG (percutaneous endoscopic gastrostomy). This can be explained by the adherence and stretching on the basal CN by VS.^22^,^25^ Hence, the low post operative KPS (76.3±15.4 SD) and 81% with GCS 13-15 in VS group. Post-operative complications in our meningiomas group is in accordant with literature. Voss et al. reported 5% (2/40) CPA meningiomas cases post operatively requiring PEG tube and tracheostomy each.^26^ Similar complications were reported by Agarwal et al.^9^ Post-operative KPS 88.6±10.8 SD and GCS 13-15 of 97.3% in accordance with existing literature.

Amir S et al. in 2019 from Islamabad reported most common complication, CSF leak (15.5%) in patients undergone suboccipital craniectomy in CPA lesion.^27^ Meta-analysis by Wu J et al. demonstrate low frequency of CSF leak in RS compared to other approaches.^28^ To our surprise none of our patients post operatively reported CSF leak which is the evidence of emerging excellence in the field of skull base surgery in Pakistan. In our study only 13.5 % (5) meningiomas and none VS cases required CSF diversion post operatively. Pirouzmand F et al. reported 35.8 % of VS cases required post op shunting. It is suggested that removing mass obviates the need of CSF diversion.^29^ But post-operative edema in a small posterior fossa compartment can cause the blockage of CSF outflow.

### Limitations:

Our study is retrospective study so there can be bias in data collection. This is a single center study and also our center does not routinely use intraoperative neuromonitoring for CPA SOL that may also limit our study results. The patients involved in the study are operated by single surgical team that may also behave as limitation. During surgery tumor were taken out in piecemeal fashion hence there exact gross sizes couldn’t be measured. Also, the detailed histopathological reports are furnished upon request and, it financially burdens the patient’s family, hence they limited our histopathological correlation. Moreover, the data of the tumor adherence to the major vasculature was inadequate hence excluded from the study. Study was conducted in public sector so there was lack of long-term follow-up which also limited our correlation with the long-term outcomes.

## CONCLUSION

The distribution of Pakistani population is suggestive of a lower mean age as compared to the mean age documented in existing literature implying early development of symptoms and aggressive cerebellopontine angle neoplasm behavior in the native population. Gross total resection within safe limits ensures improved postoperative outcomes using retrosigmoid approach for cerebellopontine schwannomas and cerebellopontine angle meningiomas. Schwannomas were more likely to have postoperative facial nerve weakness and loss of gag reflex in comparison to meningiomas. However, there are no significant differences between Schwannomas and meningiomas in terms of tumor consistency, vascularity and extent of resection.

### Authors’ Contributions:

**TIK:** Supervision, concept and design of the work, critical review of the manuscript.

**AG and MS:** Data interpretation and critical review of the manuscript.

**FIK and HMQ:** Data acquisition and data analysis, drafted the manuscript.

All authors agree to the final approval of the version to be published and be accountable for all aspects of the work.

## References

[1] Kankane VK, Warade AC, Misra BK (2019). Nonvestibular schwannoma tumors in the cerebellopontine angle:a single-surgeon experience. Asian J Neurosurg.

[2] Hassan MS, Koksal A (2023). Cerebellopontine angle meningioma in a patient presented as acute cerebrovascular accident. Ann Med Surg (Lond).

[3] Samii M, Gerganov VM (2012). Tumors of the cerebellopontine angle. Handb Clin Neurol.

[4] Adib SD, Tatagiba M (2019). Surgical management of collision-tumors between vestibular schwannoma and meningioma in the cerebellopontine angle in patients with neurofibromatosis type 2. Acta Neurochir (Wien).

[5] Mkrtchyan N, Alciato L, Kalamarides M, Bernardeschi D, Sterkers O, Bernat I (2022). Hearing recovery after surgical resection of non-vestibular schwannoma cerebellopontine angle tumors. Eur Arch Otorhinolaryngol.

[6] Moffat DA, Saunders JE, Mcelveen JT, Mcferran DJ, Hardy DG (1993). Unusual cerebello-pontine angle tumours. J Laryngol Otol.

[7] Kane AJ, Sughrue ME, Rutkowski MJ, Berger MS, Mcdermott MW, Parsa AT (2011). Clinical and surgical considerations for cerebellopontine angle meningiomas. J Clin Neurosci.

[8] Memari F, Hassannia F, Abtahi SH (2015). Surgical outcomes of cerebellopontine angle tumors in 50 cases. Iran J Orl.

[9] Agarwal V, Babu R, Grier J, Adogwa O, Back A, Friedman Ah (2013). Cerebellopontine angle meningiomas:postoperative outcomes in a modern cohort. Neurosurg Focus.

[10] Koo M, Lai JT, Yang EY, Liu TC, Hwang JH (2018). Incidence of vestibular schwannoma in taiwan from 2001 to 2012:a population-based national health insurance study. Ann Orl.

[11] Babu R, Sharma R, Bagley JH, Hatef J, Friedman AH, Adamson C (2013). Vestibular schwannomas in the modern era:epidemiology, treatment trends, and disparities in management. J Neurosurg.

[12] Spena G, Sorrentino T, Altieri R, Zinis Lr, Stefini R, Panciani PP (2018). Early-career surgical practice for cerebellopontine angle tumors in the era of radiosurgery. J Neurol Surg B Skull Base.

[13] Carlson ML, Marston AP, Glasgow AE, Habermann EB, Sweeney AD, Link MJ (2016). Racial differences in vestibular schwannoma. Laryngoscope.

[14] Magill ST, Rick JW, Chen WC, Haase DA, Raleigh DR, Aghi MK (2018). Petrous face meningiomas:classification, clinical syndromes, and surgical outcomes. World Neurosurg.

[15] Qi ZY, Shao C, Huang YL, Hui GZ, Zhou YX, Wang Z (2013). Reproductive and exogenous hormone factors in relation to risk of meningioma in women:a meta-analysis. Plos One.

[16] Ogasawara C, Philbrick BD, Adamson DC (2021). Meningioma:a review of epidemiology, pathology, diagnosis, treatment, and future directions. Biomedicines.

[17] Bonneville F, Savatovsky J, Chiras J (2007). Imaging of cerebellopontine angle lesions:an update. Part 1:enhancing extra-axial lesions. Eur Radiol.

[18] Watts J, Box G, Galvin A, Brotchie P, Trost N, Sutherland T (2014). Magnetic resonance imaging of meningiomas:a pictorial review. Insights Imaging.

[19] Gosal JS, Behari S, Joseph J, Jaiswal AK, Sardhara JC, Iqbal M (2018). Surgical excision of large-to-giant petroclival meningiomas focusing on the middle fossa approaches:the lessons learnt. Neurol India.

[20] Inskip PD, Tarone RE, Hatch EE, Wilcosky TC, Selker RG, Fine HA (2003). Laterality of brain tumors. Neuroepidemiology.

[21] Rizk AR, Adam A, Gugel I, Schittenhelm J, Tatagiba M, Ebner Fh (2017). Implications of vestibular schwannoma consistency:analysis of 140 cases regarding radiologic and clinical features. World Neurosurg.

[22] Wanibuchi M, Fukushima T, Friedman AH, Watanabe K, Akiyama Y, Mikami T (2014). Hearing preservation surgery for vestibular schwannomas via the retrosigmoid transmeatal approach:surgical tips. Neurosurg Rev.

[23] Little KM, Friedman AH, Sampson JH, Wanibuchi M, Fukushima T (2005). Surgical management of petroclival meningiomas:defining resection goals based on risk of neurological morbidity and tumor recurrence rates in 137 patients. Neurosurg.

[24] Sauvigny T, Ricklefs FL, Hoffmann L, Schwarz R, Westphal M, Schmidt NO (2020). Features of tumor texture influence surgery and outcome in intracranial meningioma. Neurooncol Adv.

[25] Awan MS, Qureshi HU, Sheikh AA, Ali MM (2001). Vestibular schwannomas:clinical presentation, management and outcome. J Pak Med Assoc.

[26] Voss NF, Vrionis FD, Heilman CB, Robertson JH (2000). Meningiomas of the cerebellopontine angle. Surg Neurol.

[27] Sohail Amir, Aurang Zeb (2019). Cerebellopontine angle tumors:a clinical study in a tertiary care hospital. Khyber J Med Sci.

[28] Jun W, Gao YL, Yu HG, Huang QL, Long XQ, Liu GH (2020). Comparison of translabyrinthine and retrosigmoid approach for treating vestibular schwannoma:A meta-analysis. Clin Neurol Neurosurg.

[29] Pirouzmand F, Tator Ch, Rutka J (2001). Management of hydrocephalus associated with vestibular schwannoma and other cerebellopontine angle tumors. Neurosurg.

